# Charles Darwin and the Theory of Natural Selection

**Published:** 1899-09

**Authors:** 


### Reviews of Books.

**Charles Darwin and the Theory of Natural Selection.** By EDWARD B. POULTON, F.R.S. Pp. 224. London: Cassell and Company, Limited. 1896.

An American naturalist has well described this little book as one of the minor classics of evolutional literature. Admirable in its clearness and lucidity, admirable in the evolution of its evolutionary theme, admirable in its portrayal not only of the central figure but of his environment, it is above all admirable in the skill with which it depicts the combined insight and patience, breadth of view and minute attention to detail, strength of intellect and lovable simpleness of character, perseverance and strength of will in one who for long had every excuse to be a confirmed invalid, which characterise Charles Darwin. Professor Poulton's book should appeal not only to biologists, but to all who would gain strength and inspiration from the life-work of a man of whom England may well be proud.

Darwin's relations to Mr. Alfred Russel Wallace afford a lesson which smaller men might do well to lay to heart. Inspired by the work of Malthus on Population, Darwin brooded over the subject of competition and struggle for existence for twenty years; he pondered over the store of facts he had amassed during the voyage of *The Beagle*, ransacked the literature of natural history, tested everything by observation and experiment, discussed the developing theory of natural selection with a few chosen friends such as Lyell and Sir Joseph Hooker, and still refrained from publishing. Mr. Wallace, inspired by the germinal idea derived from the same source, thought out the whole of his theory in a couple of hours, and completed his essay in three days. It was sent to Darwin with the request that if he thought well of it he should send it to Lyell for perusal. Through the urgent persuasion of Lyell and Hooker, Darwin was induced to publish, at the same time as Mr. Wallace's essay, an abstract of his views, together with a letter to Asa Gray in which they were summarised. "I was," he says, "at first unwilling to consent, as I thought Mr. Wallace might consider my doing so unjustifiable, for I did not then know how generous and noble was his disposition." May we not regard with pride these two great men—for Mr. Wallace is great with another greatness—for whom such an incident was not a nucleus of jealousy and bitterness, for it laid the foundations of a life-long friendship, strengthened by mutual confidence and esteem?

Alike to those who know their Darwinian literature and have read Mr. Francis Darwin's worthy life of his father, and to those who desire a short but sufficient sketch in clear perspective, we recommend Professor Poulton's book, confident that they will not be disappointed and that they will endorse our high opinion of its merits.

